# Comparative aspects of targeted sentinel lymph node mapping in veterinary and human medicine: opportunities for future research

**DOI:** 10.3389/fmed.2024.1342456

**Published:** 2024-04-03

**Authors:** Michelle L. Oblak, Hui Yu Lu, Ann S. Ram, Charly McKenna

**Affiliations:** ^1^Department of Clinical Studies, Ontario Veterinary College, University of Guelph, Guelph, ON, Canada; ^2^Department of Biomedical Sciences, Ontario Veterinary College, University of Guelph, Guelph, ON, Canada

**Keywords:** sentinel lymph node mapping, indocyanine green, comparative medicine, companion animals, ^99m^Tc-tilmanocept, OTL38, panitumumab-IRDye800CW

## Abstract

There is a significant overlap in the genetic, metabolic and epigenetic alterations between human and companion animal cancers, including those of the oral cavity, breast, bladder, skin, lungs and pancreas. In many cancer types, the identification and removal of affected lymph nodes are essential for accurate cancer management, including treatment and prognosis. Historically, lymphadenectomy and subsequent radical resection based on regional anatomy, palpation and lymph node aspirates were considered sufficient; however, modern approaches with sentinel lymph node mapping (SLN) mapping have increased the accuracy of surgical decision-making. Preoperative and intraoperative SLN mapping techniques in veterinary patients parallel those used in human medicine. While many of these techniques are highly successful, the main challenges with current methodologies are their sensitivity and specificity for the presence of cancer, which can be overcome via precision medicine and targeted SLN mapping agents. Given the large population of dogs and cats with cancer, the crossover of knowledge between species can help to deepen our understanding of many of these cancers and can be useful in evaluating new drugs and/or therapies. In this review, we discuss SLN mapping techniques in veterinary medicine and the concept of precision medicine as it relates to targeted SLN mapping imaging agents. The large number of companion animals affected by cancer is an underutilized resource to bridge the translational gap and we aim to provide a reference for the use of dogs and cats as a comparative model for human SLN mapping.

## Introduction

There are limitations to using laboratory animals to advance our understanding of cancer and evaluate new drugs and/or therapies. With their homogenous environments, a smaller overall size, and induced tumors—laboratory animals are unable to mirror the complex genetic, metabolic and epigenetic alterations associated with cancer (1, 2). Like humans, naturally occurring cancer in companion animals (dogs and cats) is one of the leading causes of death (1–5). Living in the same environments, humans and companion animals share many of the same environmental and socioeconomic factors that can predispose cancer development. These risk factors include, but are not limited to, air pollution, pesticides, age and obesity (1–4). The large number of companion animals affected by cancer is an underutilized resource to bridge the translational gap. Naturally occurring cancer in dogs and cats can provide an intermediary step between artificially induced laboratory models and human patients.

Dogs, cats and humans develop cancers with similar presentation, common phenotypes, clinical pathologies (receptors, copy number), progression and treatment response (1–5). There are many examples of comparative cancers including oral cavity squamous cell carcinoma, mammary (breast) cancer, bladder and prostate cancer, lymphoma, melanoma and pulmonary adenocarcinoma (1–5). With the relatively short life span of companion animals, in comparison to humans, cancer progresses quicker, allowing for timely assessment of novel therapies and continuity of long-term data; endpoints [disease-free interval (DFI) and median survival time (MST) (i.e., 1–2 years vs. 5–10 years) (1–3)]. In addition, identical diagnostic and imaging modalities can streamline the interpretation and comparison of results. There is also relative ease for companion animal trials as regulations are more flexible than human medicine, and the overall cost is much lower (1, 4). Moreover, increasing sentiments of companion animals as valuable household members and a desire to extend their lifespans have led to an increasing demand for improved human-level care and advanced techniques through veterinary clinical research (5–8). Due to all of these factors, there has been an increasing interest in including companion animals in the therapy development pathway (1–5, 9).

Surgical excision of a tumor(s) and lymphadenectomy is the first-line treatment for many cancer types. Nodal involvement has a significant bearing on tumor staging, treatment, and prognosis. The sentinel lymph node (SLN) is a tumor’s primary or first draining lymph node(s). Understanding the SLN allows for decreased surgical dissection, more accurate lymph node extirpation, and better overall outcomes (10, 11). Comparative cancers in dogs, cats, and humans tend to have similar lymphatic metastasis rates, making them a good model for comparison. While there are many similarities in the lymphatic anatomy and function between species, several notable differences exist. Firstly, dogs and cats have an overall fewer number of lymph nodes. Dogs and cats have only 1–2 axillary lymph nodes per side, while it is estimated that there are 20–30 axillary lymph nodes in the human body (12, 13). Secondly, unique anatomical species differences can influence the identity of the SLN. For example, dogs and cats have 8–10 mammary glands evenly distributed on their ventral abdomen, while humans have 2 mammary glands closer to the forearms. In over 90% of clinically node-negative human breast cancer patients, SLN mapping and biopsies are regarded as crucial diagnostic tests due to the large number of axillary lymph nodes (10, 11). In dogs with mammary cancer, the SLN was determined to be the axillary or accessory axillary lymph node(s) when the tumor was in the first, second, or third mammary gland and the superficial inguinal and medial iliac lymph node(s) when the tumor was in the fourth mammary gland (14). Interestingly, there seems to be wide variation and a lack of clear pattern for the tumors located at the fifth or inguinal mammary gland (14–17). Therefore, in companion animals, the SLN could be the axillary or superficial inguinal lymph node, depending on the location of the affected mammary gland, compared to a human patient with breast cancer where the SLN is typically the axillary lymph node (10, 11, 16, 18).

This review will focus on comparative aspects of SLN mapping as a reference for the potential inclusion of companion animals in future developmental pipelines for targeted SLN mapping agents.

## Sentinel lymph node mapping techniques in veterinary medicine

While SLN mapping has become the standard of care for many human tumor types, it is a relatively new concept to veterinary medicine, becoming more prevalent in the last 10–15 years. These techniques often involve a combination of preoperative and intraoperative methods. Preoperative modalities include indirect lymphography using radiography or computed tomography (CT), lymphoscintigraphy and contrast-enhanced ultrasound (CEUS). Commonly used intraoperative techniques include gross visualization following an injection of blue dyes [such as methylene blue (MB)], or near-infrared fluorescence (NIRF) imaging with fluorescent dyes such as indocyanine green (ICG).

**Figure 1:**
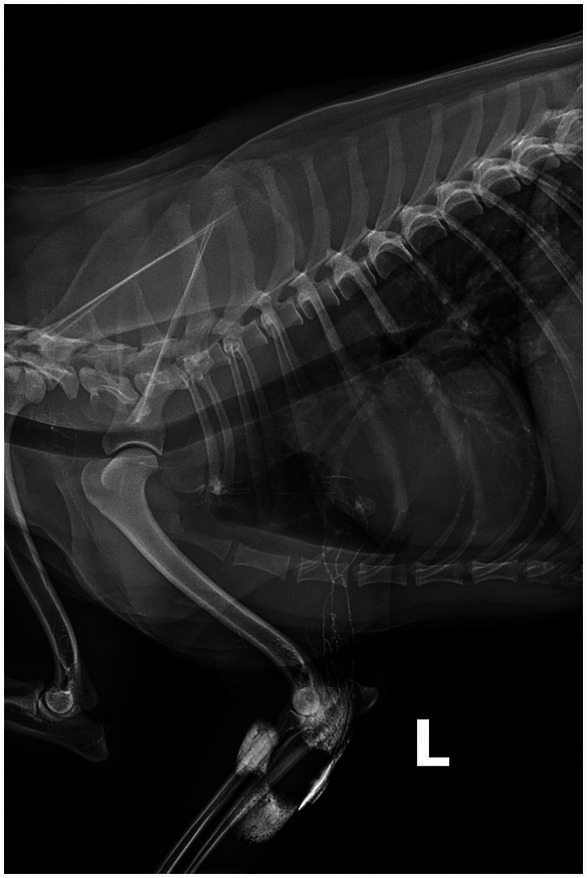
Eight-year-old spayed female dog, with a cytologically confirmed mast cell tumor and metastasis to the left prescapular lymph node. A 2 cm cutaneous mass was present along the medial aspect of the left elbow. Indirect radiographic lymphography following peritumoral intradermal 4-quadrant injection of 4 mL (total) Omnipaque 350 (GE Healthcare). Contrast medium extends from the injection site through the afferent lymphatic ducts to the left axillary and accessory axillary lymph node. Contrast within the lymphatic ducts between the left accessory axillary and axillary lymph node is also noted.

Indirect radiographic lymphography (IRL) has been successfully applied in healthy and diseased dogs. Contrast is introduced through a series (most often a quadrant pattern) of intradermal/subcutaneous injections around the tumor (Figure 1) (19, 20). Contrast agents reported in veterinary patients include both water-soluble (iopamidol, iohexol) and lipid-based (lipiodol) solutions (19, 21–23). Advantages of IRL include its excellent safety profile, low cost, simple injection technique, and widespread availability of radiography in most veterinary clinics (14, 19, 20). Furthermore, IRL can be performed in animals with sedation rather than general anesthesia (14, 20). Mayer et al. reported uniform contrast uptake in associated lymph nodes following injections of lipid-based contrast in 8 different locations (head, neck, ventral abdomen and limbs) in 16 healthy purpose-bred dogs (19). A similar study by De Bonis et al. reported an SLN detection rate of 90% using lipiodol in 23 dogs diagnosed with 26 MCTs (23). Haas et al. evaluated IRL with water-soluble contrast in 53 dogs with 59 mast cell tumors (MCT), reporting a diagnostic rate of 77.9% (20). The SLN of canine cutaneous and subcutaneous mast cell tumors of the trunk and limbs are most investigated due to the lack of agreement between the locoregional lymph node and the SLN, with disagreement rates ranging between 28 to 63% (24). In a study published by Annoni et al., where SLN mapping was performed in 80 dogs with 138 mast cell tumors, 84% originated from the trunk and limbs (25). The SLN was found to differ from the locoregional lymph node in 57% of the cases, and IRL successfully mapped the SLN in 95% of cases.

**Figure 2:**
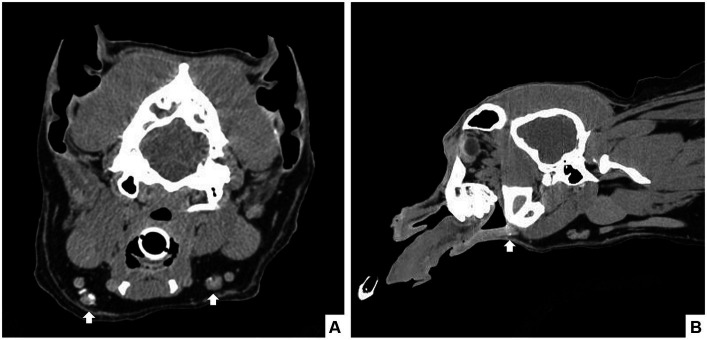
Seven-year-old castrated male dog with a histopathologically confirmed oral squamous cell carcinoma. Computed tomography revealed a mild rostral mandibular bone lesion consistent with the reported site of the mass. CT lymphangiogram with iodinated contrast injected submucosally in a 4-quadrant peritumoral injection was performed and scans were completed at 1-, 3-, and 10 min post-injection. Contrast enhancement was noted in the rostral lymphatics, with caudal extension to the bilateral mandibular lymph nodes in the 10-min scan (arrows) in transverse **(A)** and sagittal **(B)** view.

The main limitation of IRL is its lower sensitivity for SLN detection compared to CT. Despite Haas et al. reporting a high diagnostic rate of 77.9% via IRL, 20.3% of the cases were considered only partially diagnostic due to the reported visibility of the lymphatic tract but not the SLN (20). In CT lymphography (CTL), water-soluble iodinated contrast is injected directly into the lymphatics, peri or intratumorally, followed by a series of scans (Figure 2) (21). The increased resolution of CT allows for better visibility of the lymphatic vessel system, making preoperative CTL superior to radiographic lymphography (21). Sentinel lymph node detection rates using CTL are highly variable in veterinary medicine, with studies reporting rates ranging between 60 and 95%, compared to human literature where detection rates are between 95.8 and 100% (26–29). In addition, the type of contrast used for SLN mapping may affect the sensitivity of CTL. Mahieu et al. compared the use of lymphoscintigraphy and CTL in human patients with oral cancer using a lipid-based contrast and reported poor sensitivity (55%) and high negative predictive value (75%) using CTL (30). For veterinary patients, CTL is performed under general anesthesia and is typically only available in a tertiary referral hospital setting, making it a less accessible and more costly option (31). Overall limitations with both preoperative methods include the lack of real-time visual guidance and the increased exposure of hospital staff and patients to radiation.

Lymphoscintigraphy involves peritumoral injections of radiocolloids and is considered the gold standard for SLN mapping in human medicine (31–33). Lymphoscintigraphy is reported to be superior to IRL, with successful SLN detection rates of 100 and 41%, respectively. Manfredi et al. evaluated the use of preoperative lymphoscintigraphy in dogs with a malignant primary tumor (e.g., MCT, mammary adenocarcinoma, oral melanoma, thyroid carcinoma, sublingual SCC, parotid adenocarcinoma, etc.) without metastasis and the SLN was detected in 90.5% (*n* = 57/60) of tumors in 53 dogs (34). Similarly, an SLN detection rate of 91% was reported in 30 dogs with 34 MCTs that received peritumoral injections of radiocolloids and MB (35). However, the use of radiocolloids for SLN mapping is limited and is rarely applied in veterinary medicine due to its high cost and lack of accessibility (36).

Contrast-enhanced ultrasound (CEUS) is an alternative method for preoperative SLN mapping and involves peritumoral or subdermal injections of microbubbles, a high molecular weight gas that is enclosed in a lipid shell, which functions as a sonographic contrast agent (37). The use of CEUS has been documented in healthy dogs, pigs, rabbits and monkeys with success in identifying the SLN at a detection rate of 91.3–100% (38–40). Fournier et al. reported a SLN detection rate of 95.2% when CEUS was used for SLN mapping in dogs with MCT (37). While a promising alternative to other techniques, CEUS can only be performed on a superficial level and is limited by the depth of the lymphatic tracts. When comparing CEUS and ICG-NIRF, ICG-NIRF is a better technique due to its capability to identify the afferent lymphatic tracts, which are deeper within the tissues (41). In addition, radiology expertise and specialized training are required, which makes accessibility of this technique limited in veterinary practice.

**Figure 3:**
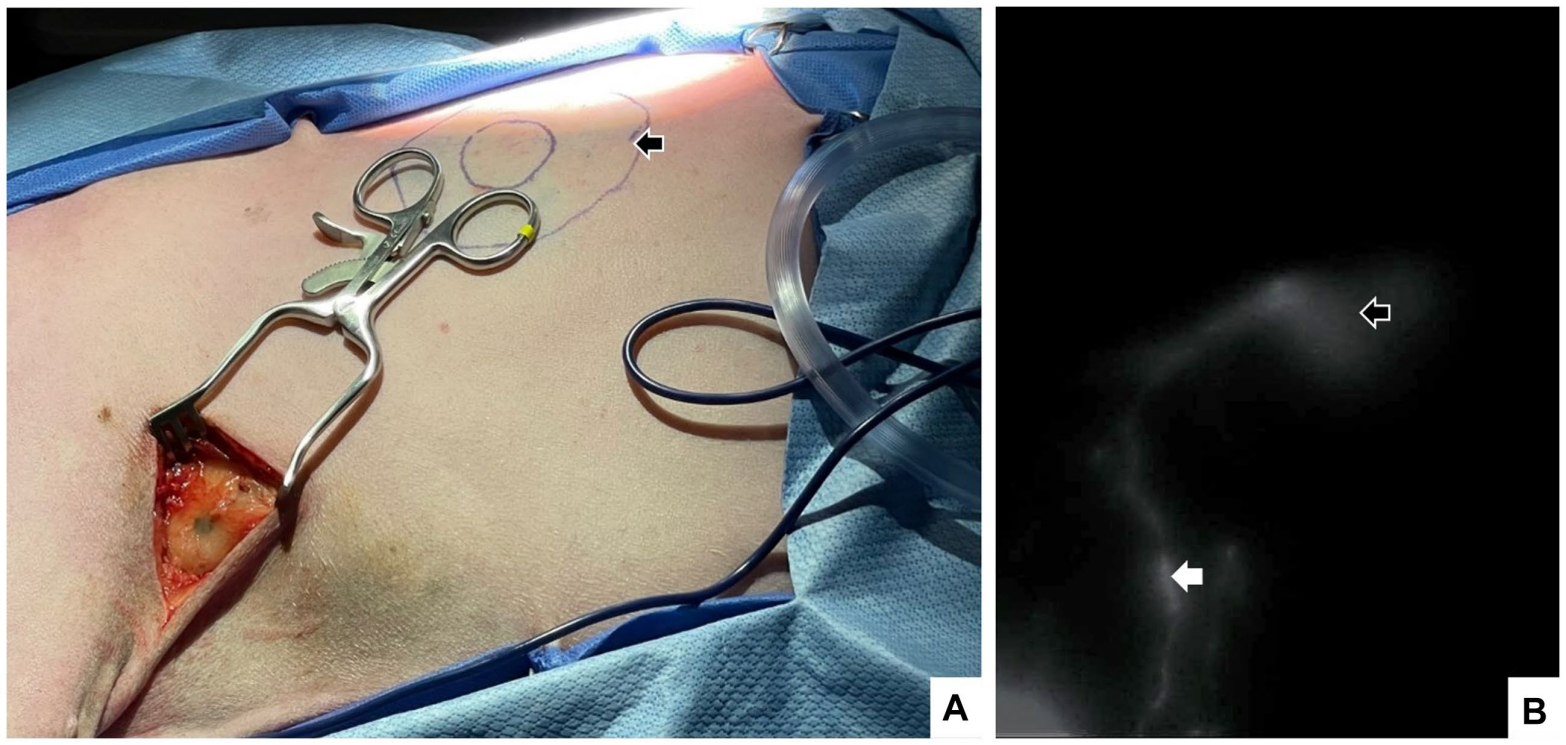
A 7-year-old spayed female dog with a cytologically confirmed mast cell tumor. The mass was 1.5 × 1.5 cm soft, mobile, flat, not well-demarcated and subcutaneous ~10 cm caudal to the left axilla on the mid-thorax. Intraoperatively, a 4-quadrant combined intradermal and subcutaneous injection of saline (1.6 mL), indocyanine green (0.5 mg, 2.5 mg/mL; Seaford Pharmaceuticals Inc) and methylene blue (2 mg, 10 mg/mL; Omega) was performed (indicated by black arrow). Lymph node dissection in the axillary region confirmed the presence of blue dye **(A)**, and near-infrared fluorescence imaging documented the injection sites (open white arrow) lymphatic tract and the left accessory axillary lymph nodes (white arrow) **(B)**.

Intraoperative SLN mapping using blue dyes, ICG, or other agents provides real-time visual guidance. Traditionally, blue dye is injected peritumorally in four quadrants surrounding the tumor, and the SLN is located based on gross visualization of blue staining (Figure 3). Using blue dye alone has been most frequently reported in human breast cancer patients, with a high identification rate of 89–91% (42, 43). In contrast to human literature, SLN detection rates in veterinary medicine are highly variable when blue dye is used alone. Ferrari et al. reported an SLN identification rate of 91% in 34 dogs with MCTs, while Wan et al. reported an SLN detection rate of 50.8% in dogs with oral cancer (22, 35). Methylene blue (MB) is a commonly reported blue dye as it is widely available and cost-effective; however, the use of MB alone heavily relies on the knowledge of the location of the SLN with respect to the tumor as blue staining is not easily visible percutaneously (42–44). Given that the success of SLN detection using MB alone is highly variable, recent literature has recommended either a shift from using MB entirely, combining MB with ICG-NIRF, or combining preoperative indirect lymphography using CT with intraoperative MB-ICG/NIRF to maximize SLN detection rates (22, 45, 46).

Compared to MB alone, ICG-NIRF has a higher SLN detection rate, greater tissue penetration, and better visualization of the afferent lymphatic tracts (22, 41, 47). Despite NIRF-ICG being a more expensive modality to MB, it is known to be less invasive, cost-effective, and has a higher sensitivity and specificity compared to lymphoscintigraphy in human literature (48). Arz et al. reported success in identifying SLNs using ICG-NIRF as small as 0.9 cm in a cat diagnosed with a low-grade MCT in the left buccal region. Indocyanine green also has an excellent safety profile and has been demonstrated to have no significant side effects in companion animals (49). Applications have included SLN mapping of the oral, head and neck region, caudal abdomen and limbs in healthy and diseased dogs and head and neck region, trunk, perineum, and limbs in healthy and diseased cats (Figure 3) (22, 29, 41, 46, 47, 50). As a result, this technique is gaining popularity in the veterinary field; however, limitations include the accessibility of NIRF equipment and a lack of protocol standardization.

## Targeted lymph node mapping agents

**Figure 4:**
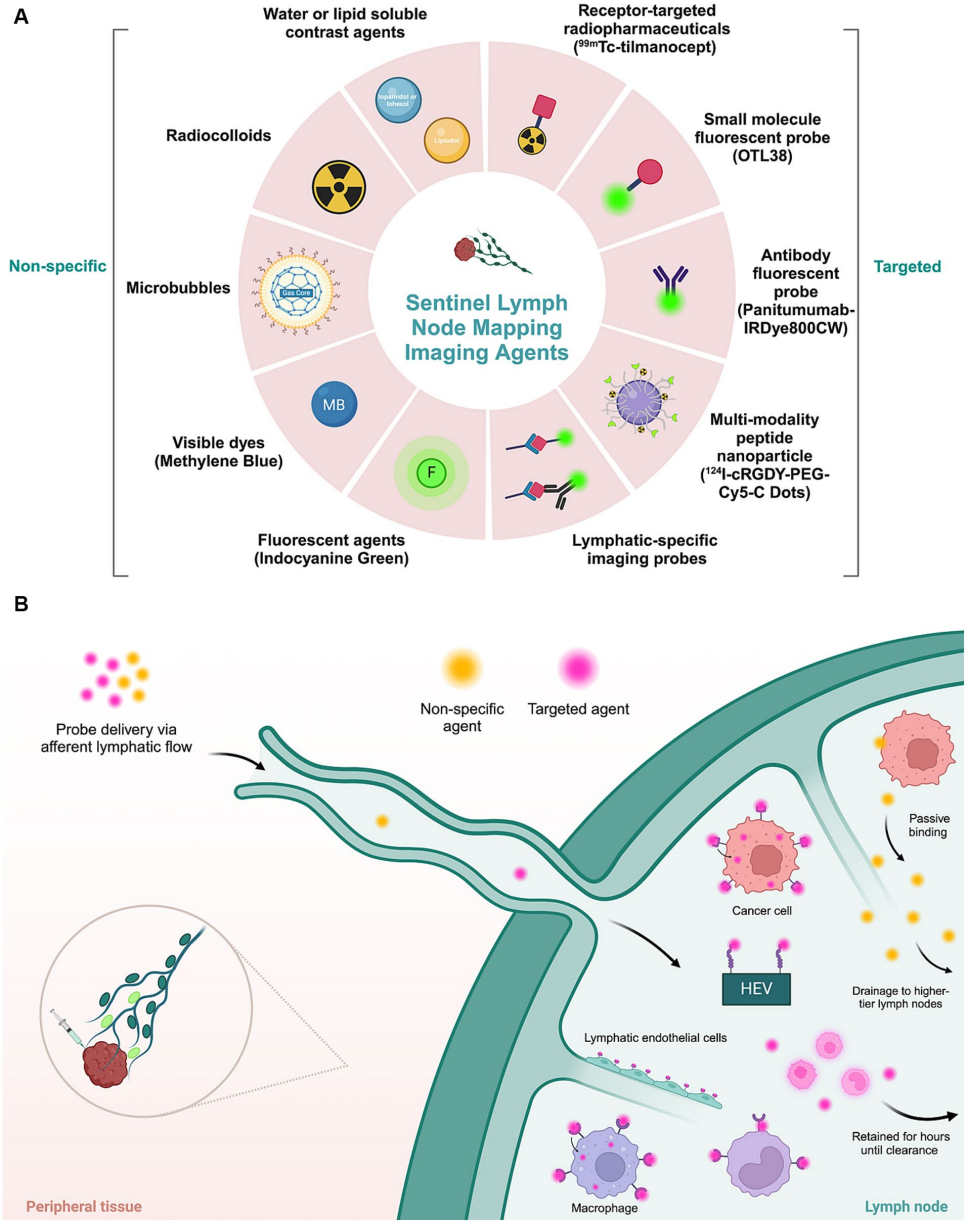
Imaging agents applied in sentinel lymph node mapping. **(A)** Molecular non-specific contrast agents and targeted moieties used for sentinel lymph node mapping. **(B)** Schematic representation of the mechanism of action of different types of imaging agents. Non-targeted agents passively accumulate in lymphatic tissue. Targeted agents, consisting of a molecular contrast agent or dye conjugated to a targeting moiety, actively accumulate in tumor or lymphatic cells by recognition of a specific biomarker expressed by tumor cells or lymphatic cells. HEV, high endothelial venules.

An optimal SLN mapping imaging agent enables rapid clearance from the injection site, has increased uptake and retention within the SLN, and has low drainage to higher-tier lymph nodes (51) (Figure 4). The previously mentioned techniques for SLN mapping employ passive strategies. These preoperative and intraoperative imaging modalities exhibit unsatisfactory sensitivity and specificity rates, decreasing the fidelity of these resections (52).

This pitfall can be overcome by developing novel ligand-receptor or antigen–antibody binding imaging probes (51). Theoretically, any imaging agent targeting tumor cells can detect both primary tumors and metastatic lymph nodes (53). In recent years, the concept of precision medicine has had unparalleled growth in human oncology; however, it is relatively new in veterinary oncology. While precision medicine encompasses many elements, particularly valuable for SLN mapping is the identification of genomic markers and tumor characteristics.

Several targeted imaging agents have been developed for biomarkers expressed in human cancers and many of these are undergoing clinical testing or have gained regulatory body approval. Dogs and cats spontaneously develop many of the same cancers as humans with similar histological subtypes and tumorigenic genes and pathways, which is fundamental for the translation of targeted therapeutics (1, 2, 9, 54). Building upon the foundation of human cancer research, comparative companion animal cancers can aid in theoretically applying or studying targeted imaging agents for SLN mapping.

### ^99m^Tc-tilmanocept (Lymphoseek)

^99m^Tc-tilmanocept (Lymphoseek) is a Food and Drug Administration (FDA) approved novel radiopharmaceutical for preoperative SLN detection in human breast, melanoma, and head and neck cancers (55–57). The mechanism of action of ^99m^Tc-tilmanocept for SLN mapping revolves around its small molecular size (16.7 kDa) and diameter (7.1 nm) which allows for rapid transportation through afferent lymphatics (55, 58). The mannose receptor (Cluster of Differentiation 206, CD206) is expressed on the surface of macrophages and reticuloendothelial cells, which are highly concentrated in lymph nodes (51, 57, 59). CD206 recognizes and binds to macromolecules with carbohydrate side chains that terminate with a mannose glycoside on ^99m^Tc-tilmanocept (51). The mannose residues on ^99m^Tc-tilmanocept bind to the receptors on the reticuloendothelial cells’ surface, enabling its accumulation and retention in the SLN for approximately 30 h (55, 58). Multiple phase I and II human clinical trials have confirmed that ^99m^Tc-tilmanocept does not migrate to the second-tier lymph nodes (55, 58).

Before targeted radiocolloids were employed, lymphatic mapping relied on standard radiocolloids, such as technetium-99 m nanocolloid (99mTc) (55, 56, 58). The radiopharmaceutical enables preoperative imaging with SPECT–CT and has slow clearance from the injection site and is passively retained in lymph nodes. This results in the inability to localize SLNs due to the overlap between area of SLNs and the injection site or insufficient uptake of the radiopharmaceutical in SLN. Compared to conventional radiocolloids and blue dyes, ^99m^Tc-tilmanocept has several advantages, including its rapid injection site clearance, high SLN identification and extraction, and low distal lymph node uptake (55, 56, 58). Limitations of ^99m^Tc-tilmanocept are attributed to its low efficacy based on tumor tissue type, as well as disadvantages typically associated with conventional radiocolloids, including ionizing radiation, accessibility, and lack of intraoperative guidance (56, 60, 61). Comparatively, ^99m^Tc-tilmanocept may have an application for preoperative SLN mapping as CD206 receptors are also expressed in tumor-associated macrophages of dogs and cats (62, 63). Stroup et al. documented the feasibility and utility of an intra-prostatic injection of gallium-68 labeled tilmanocept in four adult male purpose-bred dogs. A mean of 4 SLNs were identified per dog, and all SLN identified by the PET/CT were confirmed to be fluorescent (64).

### OTL38 (Cytalux)

OTL38 (Cytalux) is an FDA-approved NIRF imaging agent for the detection of human ovarian and lung cancer. OTL38 is a folate analog ligand conjugated to S0456, an indocyanine green-like dye which targets the folate receptor alpha (FOLR1) that is overexpressed in specific cancers (65). Combined with tumor delineation and tumor bed imaging, OTL38 has been evaluated for its ability to detect metastatic lymph nodes. To implement OTL38 for SLN mapping, the role of folate receptor beta (FOLR2) must be further elucidated due to the high rate of false positive lymph nodes (66). Activated macrophages overexpress FOLR2 and may cause false positive fluorescence (67–69). This was supported by histological confirmation in which FOLR2 staining was localized within macrophages of the subcapsular sinuses of lymph nodes, independent of lymph node micro-metastases (68, 69). Despite its high rate of false positives, OTL38 has potential use for selective SLN mapping based on factors such as cancer stage and histological subtypes that could have different lymph node metastatic rates (69). Limitations of OTL38 were noted in image interpretation errors and false positives and negatives, specifically in non-malignant regional lymph nodes (70).

The comparative use of OTL38 for SLN detection in dogs and cats is dependent on the expression of FOLR1 in companion animal cancers. In human and canine invasive transitional cell carcinoma, FOLR1 is overexpressed; therefore, the use of FOLR1-targeted therapeutics may be advantageous (2, 71). To date, OTL38 has yet to be evaluated for urothelial cancers in either humans or dogs.

Interestingly, the use of OTL38 has been evaluated in clinical lung cancer dogs despite the lack of evidence around the overexpression of FOLR1 in canine lung tumors. Keating et al. utilized OTL38 during video-assisted thoracoscopic surgery or open thoracotomy and tumor excision in 10 dogs with lung tumors (72). OTL38 identified lung tumors and margins in all dogs when administered intravenously; however, since no significant dissections were performed for lymph nodes, only 3 regional bronchial lymph nodes were identified that were also positive for metastasis. At the study institution, surgeons typically removed/sample lymph nodes that were obvious and easy to dissect rather than routinely visualize and remove all nodes during their lung cancer surgeries (72). In this study, OTL38 was also used in 3 human pulmonary adenocarcinoma patients with similar tumor and margin identification results as the dogs. No results regarding SLN mapping or lymph nodes were reported in the human patients.

### Panitumumab-IRDye800CW

Instead of switching to an alternative agent for SLN mapping, it is possible to conjugate antibodies to already proven NIRF dyes. Panitumumab is a fully humanized monoclonal Immunoglobulin G2 antibody that has a high-affinity binding to epidermal growth factor receptor (EGFR), a protein receptor overexpressed in many cancer types in dogs, cats and humans (2, 5, 9, 73–80). Panitumumab-IRDye800CW is a fluorescent antibody-dye conjugate reported to be safe and stable with a high signal-to-background ratio (73, 81). During pathological processing of lymph nodes from human patients with squamous cell carcinoma of the head and neck, panitumab-IRDye800CW strongly corresponded with EGFR-expressing metastatic areas within the lymph (82). Interestingly, fluorescence was concentrated in the periphery of the metastasis despite uniform EGFR expression within the metastatic deposit, which implies that while panitumumab-IRDye800CW can bind to lymph node metastases, it may not be the most effective choice when afferent lymphatics vessels are occluded by the growing tumor mass (82). Furthermore, as the mechanism of action is yet to be elucidated, it is hypothesized that panitumumab-IRDye800CW accumulates in the tumor and proceeds to drain non-specifically to the regional lymph node basin in a stepwise manner and, therefore metastatic SLN are more likely to retain the dye due to the large size of the tracer (81). In 27 humans with oral squamous cell carcinoma, panitumumab-IRDye800CW was infused intravenously prior to surgical resection of their primary tumor and lymphadenectomy or SLN biopsy (81). Krishan et al. reported the preferential localization of panitumumab-IRDye800CW in the metastatic and sentinel lymph nodes (81). Limitations of antibody-based imaging agents include poor tumor tissue and cellular extravasation due to their large molecular size and high production cost (83–85). The use of a fluorescent antibody-dye conjugate, such as panitumumab-IRDye800CW, has yet to be described in veterinary literature. Imaging agents that target EGFR have the potential to be applied to companion animal cancers and could be considered for SLN mapping in canine bladder cancer (3, 74), feline oral squamous cell carcinomas (9, 75–77), canine and feline mammary carcinomas (9, 78, 79), and canine osteosarcoma (9, 77, 80).

### ^124^I-cRGDY-PEG-Cy5-C dots

Ultrasmall organic–inorganic (silica) hybrid nanoparticles (Cornell dots or c-dots) have been developed to overcome the typical limitations associated with radiocolloids and near-infrared dyes. ^124^I-cRGDY-PEG-Cy5-C dots have recently gained FDA approval and is a fluorescent and integrin-targeting agent that covalently encapsulates a Cy5 dye (86). The surface of ^124^I-cRGDY-PEG-Cy5-C dots contains peptide cyclo-(Arg-Gly-Asp-Tyr) which detects integrin α_ν_β_3_ that is overexpressed in endothelial cells involved in the angiogenesis and vascular remodeling of tumor cells (87). Due to the small size, uniform delivery into nodes and other metastatic deposits, selective uptake and retention, encapsulation of the dye for ideal optical properties, and versatility, ^124^I-cRGDY-PEG-Cy5-C dots are well-suited for SLN mapping via positron emission tomography (PET) and intraoperatively with fluorescence as a dual-modality imaging (87, 88) In human studies of breast, melanoma and head and neck cancers, it has been reported that ^124^I-cRGDY-PEG-Cy5-C has high detection sensitivity of metastatic and could specifically discriminate between metastatic tumors infiltration and inflammatory responses (87, 88). ^24^I-cRGDY-PEG-Cy5-C dots utilize the C dot construct to improve depth resolution and increase the sensitivity of SLN identification in low accessibility nodal regions overcoming limitations of radiocolloids and small-molecule dyes (89, 90). These strengths exemplify the potential that novel technologies such as nanoparticles or multi-modality probes have in the future of this field (89, 90).

Comparatively, while there are no studies utilizing ^124^I-cRGDY-PEG-Cy5-C dots in companion animals, there are other integrin α_ν_β_3_ targeted imaging agents which have shown promising results in dogs and cats (91–93). Integrin α_ν_β_3_ is expressed in normal epithelia of the small intestine, kidney, lung, and spleen but is absent in the vascular tissue of dogs (94). Regarding cancer, integrin α_ν_β_3_ is reported to be highly expressed in canine cutaneous and oral melanomas (95). Favril et al. reported that DA364, a near-infrared imaging agent that targets integrins through the RGD peptide, was able to accumulate in a range of superficial solid tumors from dogs, which included mast cell tumors, mammary gland adenocarcinoma, soft tissue sarcoma, osteosarcoma, and cutaneous melanoma (91). Lymph node imaging was also performed with DA364 having an efficacy for detecting metastasis in a small number of regional lymph nodes (91). The population for this study was limited (*n* = 24), and a larger population needs to be assessed prior to any definitive conclusions.

### Lymphatic-specific imaging probes

Nodal metastasis is often microscopic; therefore, it can be ambitious to apply tumor-targeting imaging agents for SLN mapping (53). A viable alternative is using lymphangiogenesis (growth of the lymphatic vasculature) to predict metastatic lymph nodes. Lymphatic-specific markers include podoplanin, Prox-1, LYVE-1, and VEGFR-3, and a lymphatic targeting agent is developed against these markers via labeled antibodies or peptide ligands (52, 96, 97). A targeted vascular agent such as MECA-79, developed by Licha et al. allows the imaging agent to reach the target without penetrating the surrounding tissue, which is often the limiting factor for using antibodies. MECA-79 is an immunoglobulin M monoclonal antibody conjugated fluorescent dye (cyanine 7) that targets glycoproteins on the luminal side of specialized high endothelial venules in lymph nodes (98). In a murine model, as little as 0.25 nmol of MECA-79 was required per animal to enable high-sensitivity lymph node imaging and accumulation in peripheral lymph nodes (98). The novel NIRF conjugate MECA-79 has yet to be evaluated in other animal or human models.

Peptide-based imaging agents have a smaller molecular size, allowing for faster clearance than antibody-based agents. FITC-LyP-1 combines LyP-1, a cyclic 9-amino-acid cyclic peptide, and various fluorophores (53). LyP-1 is also referred to as a tumor-homing peptide as it binds to the p32 receptor that is highly expressed on lymphatic endothelium and tumor cells (52, 99). However, the binding of LyP-1 to tumor lymphatics and cells is not general for all tumors, implying that tumor cells may induce expression of LyP-1 binding protein in intratumoral lymphatic cells (99). Agents targeting lymphangiogenesis are limited in their ability to discriminate between tumoral and inflammatory lymphangiogenesis (53). As lymphangiogenesis can occur in environments with inflammation due to increased levels of lymphangiogenic factors produced by macrophages and granulocytes, studies employing these agents should be interpreted with caution. To overcome this limitation would be to employ a dual technique that combines tumor and lymphatic targeting ligands in one probe (53, 100).

For imaging agents that target lymphatic markers and lymphangiogenesis to be comparative, the expression of these targets must also be expressed in the lymphatic cells of companion animals. There are yet to be companion animal studies applied for SLN mapping using Lyp-1 imaging agents, and the expression of p23, the receptor for Lyp-1, is unknown in dogs and cats. This knowledge gap suggests that it is worthwhile to assess the expression of lymphangiogenic markers in companion animals for the future implementation of imaging agents targeting lymphatic biomarkers.

## Conclusion

Companion animals are often an integral part of our lives, sharing our environments and, subsequently, our risk factors for developing cancer. Current research has determined genomic and tumor similarities within the naturally occurring cancers between humans, dogs and cats, which can be used to develop targeted lymph node mapping agents. Cross-species use increases accessibility and enhances early adoption in clinical trials. The synergistic relationship between animal and human cancer research is vital for the future of translational research and advancements in precision medicine.

## Author contributions

MO: Conceptualization, Writing – original draft, Writing – review & editing. HL: Writing – original draft, Writing – review & editing. AR: Writing – original draft, Writing – review & editing, Conceptualization, Visualization. CM: Conceptualization, Writing – review & editing, Writing – original draft, Visualization.

## References

[1] Paoloni M Khanna C. Translation of new cancer treatments from pet dogs to humans. Nat Rev Cancer. (2008) 8:147–56. doi: 10.1038/nrc2273 18202698

[2] Oh JH Cho JY. Comparative oncology: overcoming human cancer through companion animal studies. Exp Mol Med. (2023) 55:725–34. doi: 10.1038/s12276-023-00977-3, PMID: 37009802 PMC10167357

[3] Sarver AL Makielski KM DePauw TA Schulte AJ Modiano JF. Increased risk of cancer in dogs and humans: a consequence of recent extension of lifespan beyond evolutionarily determined limitations? Aging Cancer. (2022) 3:3–19. doi: 10.1002/aac2.12046, PMID: 35993010 PMC9387675

[4] Furdos I Fazekas J Singer J Jensen-Jarolim E. Translating clinical trials from human to veterinary oncology and back. J Transl Med. (2015) 13:265. doi: 10.1186/s12967-015-0631-9, PMID: 26275615 PMC4536666

[5] Cannon CM. Cats, Cancer and comparative oncology. Vet Sci. (2015) 2:111–26. doi: 10.3390/vetsci2030111, PMID: 29061935 PMC5644631

[6] National Research Council (US) Committee on the National Needs for research in veterinary science. Critical needs for research in veterinary science. Washington, DC: National Academies Press (2005). 20669456

[7] McConnell AR Lloyd PE Humphrey BT. We are family: viewing pets as family members improves wellbeing. Anthrozoös. (2019) 32:459–70. doi: 10.1080/08927936.2019.1621516

[8] Bouma EMC Reijgwart ML Dijkstra A. Family member, best friend, child or 'Just' a pet, Owners' relationship perceptions and consequences for their cats. Int J Environ Res Public Health. (2021) 19:193. doi: 10.3390/ijerph19010193, PMID: 35010452 PMC8750854

[9] Hernández IB Kromhout JZ Teske E Hennink WE van Nimwegen SA Oliveira S. Molecular targets for anticancer therapies in companion animals and humans: what can we learn from each other? Theranostics. (2021) 11:3882–97. doi: 10.7150/thno.55760, PMID: 33664868 PMC7914358

[10] Chatterjee A Serniak N Czerniecki BJ. Sentinel lymph node biopsy in breast cancer: a work in progress. Cancer J. (2015) 21:7–10. doi: 10.1097/PPO.0000000000000090, PMID: 25611773 PMC4304410

[11] Yasmin T Numair Younis M Masood M Majeed Khan H Asgher Z Shahid A. Sentinel lymph node mapping in breast Cancer: initial experience of a multidisciplinary team. Cureus. (2022) 14:e25983. doi: 10.7759/cureus.25983, PMID: 35859965 PMC9286901

[12] Rehnblom ER Skinner OT Mickelson MA Hutcheson KD. Axillary lymphadenectomy in dogs: a description of surgical technique. Vet Comp Oncol. (2022) 20:664–8. doi: 10.1111/vco.12820, PMID: 35411711

[13] Kyriacou H Khan YS. Anatomy, shoulder and upper limb, axillary lymph nodes In: StatPearls. Treasure Island, FL: StatPearls Publishing (2023) 32644614

[14] Collivignarelli F Tamburro R Aste G Falerno I Del Signore F Simeoni F. Lymphatic drainage mapping with indirect Lymphography for canine mammary tumors. Animals (Basel). (2021) 11:1115. doi: 10.3390/ani11041115, PMID: 33924625 PMC8070006

[15] Patsikas MN Dessiris A. The lymph drainage of the mammary glands in the bitch: a lymphographic study. Part I: the 1st, 2nd, 4th and 5th mammary glands. Anat Histol Embryol. (1996) 25:131–8. doi: 10.1111/j.1439-0264.1996.tb00071 8766408

[16] Soultani C Patsikas MN Karayannopoulou M Jakovljevic S Chryssogonidis I Papazoglou L. Assessment of sentinel lymph node metastasis in canine mammary gland tumors using computed tomographic indirect lymphography. Vet Radiol Ultrasound. (2017) 58:186–96. doi: 10.1111/vru.12460, PMID: 28009075

[17] Favril S Abma E Stock E Devriendt N van Goethem B Blasi F. Fluorescence-guided surgery using indocyanine green in dogs with superficial solid tumours. Vet Rec. (2020) 187:273. doi: 10.1136/vr.105554, PMID: 32345608

[18] de Souza MCC Flecher MC Arrais FM de Sena BV Giuliano A Horta RDS. Comparison of surgical resection of axillary lymph nodes in dogs with mammary gland tumors with or without sentinel lymph node visualization with patent blue dye. Front Vet Sci. (2023) 10:1149315. doi: 10.3389/fvets.2023.1149315, PMID: 37252402 PMC10213635

[19] Mayer MN Silver TI Lowe CK Anthony JM. Radiographic lymphangiography in the dog using iodized oil. Vet Comp Oncol. (2012) 11:151–61. doi: 10.1111/j.1476-5829.2012.00334.x, PMID: 22630597

[20] Haas S Linden D Cole R Smith A Schleis S Matz B. Indirect lymphography for sentinel lymph node detection in dogs with mast cell tumors. Can Vet J. (2023) 64:142–8. PMID: 36733656 PMC9847431

[21] Rossi F Körner M Suárez J Carozzi G Meier VS Roos M. Computed tomographic-lymphography as a complementary technique for lymph node staging in dogs with malignant tumors of various sites. Vet Radiol Ultrasound. (2018) 59:155–62. doi: 10.1111/vru.12569, PMID: 29024279

[22] Wan J Oblak ML Ram A Singh A Nykamp S. Determining agreement between preoperative computed tomography lymphography and indocyanine green near infrared fluorescence intraoperative imaging for sentinel lymph node mapping in dogs with oral tumours. Vet Comp Oncol. (2021) 19:295–303. doi: 10.1111/vco.12675, PMID: 33403753

[23] De Bonis A Collivignarelli F Paolini A Falerno I Rinaldi V Tamburro R. Sentinel lymph node mapping with indirect lymphangiography for canine mast cell tumour. Vet Sci. (2022) 9:484. doi: 10.3390/vetsci9090484, PMID: 36136700 PMC9503988

[24] Worley DR. Incorporation of sentinel lymph node mapping in dogs with mast cell tumours: 20 consecutive procedures. Vet Comp Oncol. (2014) 12:215–26. doi: 10.1111/j.1476-5829.2012.00354.x 22958227

[25] Annoni M Borgonovo S Aralla M. Sentinel lymph node mapping in canine mast cell tumours using a preoperative radiographic indirect lymphography: technique description and results in 138 cases. Vet Comp Oncol. (2023) 21:469–81. doi: 10.1111/vco.12906, PMID: 37191042

[26] Motomura K Sumino H Noguchi A Horinouchi T Nakanishi K. Sentinel nodes identified by computed tomography-lymphography accurately stage the axilla in patients with breast cancer. BMC Med Imaging. (2013) 13:42. doi: 10.1186/1471-2342-13-42, PMID: 24321242 PMC4028847

[27] Grimes JA Secrest SA Northrup NC Saba CF Schmiedt CW. Indirect computed tomography lymphangiography with aqueous contrast for evaluation of sentinel lymph nodes in dogs with tumors of the head. Vet Radiol Ultrasound. (2017) 58:559–64. doi: 10.1111/vru.12514, PMID: 28543945

[28] Goldschmidt S Stewart N Ober C Bell C Wolf-Ringwall A Kent M. Contrast-enhanced and indirect computed tomography lymphangiography accurately identifies the cervical lymphocenter at risk for metastasis in pet dogs with spontaneously occurring oral neoplasia. PLoS One. (2023) 18:e0282500. doi: 10.1371/journal.pone.0282500, PMID: 36862650 PMC9980747

[29] Ferraris EI Olimpo M Giacobino D Manassero L Iussich S Lardone E. Sentinel lymph node mapping with computed tomography lymphography for mast cell tumours and a comparison between regional and sentinel lymph node histological status: sixty-two cases. Vet Comp Oncol. (2023) 21:208–20. doi: 10.1111/vco.12878 36635868

[30] Mahieu R Donders DNV Dankbaar JW de Bree R de Keizer B. CT Lymphography using Lipiodol® for sentinel lymph node biopsy in early-stage Oral Cancer. J Clin Med. (2022) 11:5129. doi: 10.3390/jcm11175129, PMID: 36079061 PMC9456579

[31] Beer P Chiti LE Nolff MC. The role of sentinel node mapping and lymphadenectomies in veterinary surgical oncology. Lymphatics. (2023) 1:2–18. doi: 10.3390/lymphatics1010002

[32] Vu HN O’Connor PF Shoemaker RR Wan W Fratkin MJ Bear HD. Intraoperative injection of Radiocolloid for sentinel node biopsy in breast Cancer. J Nucl Med Technol. (2013) 41:263–7. doi: 10.2967/jnmt.113.129460, PMID: 24231723

[33] Ranzenberger LR Pai RB. Lymphoscintigraphy In: StatPearls. Treasure Island, FL: StatPearls Publishing (2024) 33085360

[34] Manfredi M De Zani D Chiti LE Ferrari R Stefanello D Giudice C. Preoperative planar lymphoscintigraphy allows for sentinel lymph node detection in 51 dogs improving staging accuracy: feasibility and pitfalls. Vet Radiol Ultrasound. (2021) 62:602–9. doi: 10.1111/vru.12995, PMID: 34131982 PMC8518895

[35] Ferrari R Chiti LE Manfredi M Ravasio G De Zani D Zani DD. Biopsy of sentinel lymph nodes after injection of methylene blue and lymphoscintigraphic guidance in 30 dogs with mast cell tumors. Vet Surg. (2020) 49:1099–108. doi: 10.1111/vsu.13483

[36] Hlusko KC Cole R Tillson DM Boothe HW Almond G Coggeshall WS. Sentinel lymph node detection differs when comparing lymphoscintigraphy to lymphography using water soluble iodinated contrast medium and digital radiography in dogs. Vet Radiol Ultrasound. (2020) 61:659–66. doi: 10.1111/vru.12908, PMID: 32929849

[37] Fournier Q Thierry F Longo M Malbon A Cazzini P Bisson J. Contrast-enhanced ultrasound for sentinel lymph node mapping in the routine staging of canine mast cell tumours: a feasibility study. Vet Comp Oncol. (2021) 19:451–62. doi: 10.1111/vco.12647, PMID: 32840038

[38] Goldberg BB Merton DA Liu JB Murphy G Forsberg F. Contrast-enhanced sonographic imaging of lymphatic channels and sentinel lymph nodes. J Ultrasound Med. (2005) 24:953–65. doi: 10.7863/jum.2005.24.7.953, PMID: 15972710

[39] Gelb HR Freeman LJ Rohleder JJ Snyder PW. Feasibility of contrast-enhanced ultrasound-guided biopsy of sentinel lymph nodes in dogs. Vet Radiol Ultrasound. (2010) 51:628–33. doi: 10.1111/j.1740-8261.2010.01712.x, PMID: 21158235

[40] Wang Y Cheng Z Li J Tang J. Gray-scale contrast-enhanced ultrasonography in detecting sentinel lymph nodes: an animal study. Eur J Radiol. (2010) 74:e55–9. doi: 10.1016/j.ejrad.2009.03.063, PMID: 19423261

[41] Favril S Stock E Hernot S Hesta M Polis I Vanderperren K. Sentinel lymph node mapping by near-infrared fluorescence imaging and contrast-enhanced ultrasound in healthy dogs. Vet Comp Oncol. (2019) 17:89–98. doi: 10.1111/vco.12449, PMID: 30311430

[42] Guo J Yang H Wang S Cao Y Liu M Xie F. Comparison of sentinel lymph node biopsy guided by indocyanine green, blue dye, and their combination in breast cancer patients: a prospective cohort study. World J Surg Oncol. (2017) 15:196. doi: 10.1186/s12957-017-1264-7, PMID: 29096643 PMC5667473

[43] Li J Chen X Qi M Li Y. Sentinel lymph node biopsy mapped with methylene blue dye alone in patients with breast cancer: a systematic review and meta-analysis. PLoS One. (2018) 13:e0204364. doi: 10.1371/journal.pone.0204364, PMID: 30235340 PMC6147575

[44] Xu YL Liu XJ Zhu Y Lu H. Preoperative localization of sentinel lymph nodes using percutaneous contrast-enhanced ultrasonography in patients with breast cancer. Gland Surg. (2022) 11:369–77. doi: 10.21037/gs-22-10, PMID: 35284303 PMC8899426

[45] Hirche C Murawa D Mohr Z Kneif S Hünerbein M. ICG fluorescence-guided sentinel node biopsy for axillary nodal staging in breast cancer. Breast Cancer Res Treat. (2010) 121:373–8. doi: 10.1007/s10549-010-0760-z, PMID: 20140704

[46] Lu HY McKenna C Ram A Oblak ML. Effect of volume and methylene blue on fluorescence intensity and transit of indocyanine green for sentinel lymph node mapping in a simulated feline tumor model. Am J Vet Res. (2023) 84(11):ajvr.23.07.0168:1–7. doi: 10.2460/ajvr.23.07.0168 37683839

[47] Townsend KL Milovancev M Bracha S. Feasibility of near-infrared fluorescence imaging for sentinel lymph node evaluation of the oral cavity in healthy dogs. Am J Vet Res. (2018) 79:995–1000. doi: 10.2460/ajvr.79.9.995, PMID: 30153060

[48] Akita S Mitsukawa N Kazama T Kuriyama M Kubota Y Omori N. Comparison of lymphoscintigraphy and indocyanine green lymphography for the diagnosis of extremity lymphoedema. J Plast Reconstr Aesthet Surg. (2013) 66:792–8. doi: 10.1016/j.bjps.2013.02.023, PMID: 23523168

[49] Arz R Chiti LE Krudewig C Grieco V Meier V Fejös C. Lymph node metastasis in feline cutaneous low-grade mast cell tumours. J Feline Med Surg. (2023) 25:1098612X2211384. doi: 10.1177/1098612X221138468, PMID: 36638145 PMC10812050

[50] Chiti LE Gariboldi EM Stefanello D De Zani D Grieco V Nolff MC. Sentinel lymph node mapping and biopsy in cats with solid malignancies: an explorative study. Animals (Basel). (2022) 12:3116. doi: 10.3390/ani12223116, PMID: 36428344 PMC9686746

[51] Wallace AM Hoh CK Vera DR Darrah DD Schulteis G. Lymphoseek: a molecular radiopharmaceutical for sentinel node detection. Ann Surg Oncol. (2003) 10:531–8. doi: 10.1245/ASO.2003.07.012, PMID: 12794019

[52] Ji H Hu C Yang X Liu Y Ji G Ge S. Lymph node metastasis in cancer progression: molecular mechanisms, clinical significance and therapeutic interventions. Sig Transduct Target Ther. (2023) 8:367. doi: 10.1038/s41392-023-01576-4, PMID: 37752146 PMC10522642

[53] Niu G Chen X. Lymphatic imaging: focus on imaging probes. Theranostics. (2015) 5:686–97. doi: 10.7150/thno.11862, PMID: 25897334 PMC4402493

[54] LeBlanc AK Mazcko CN. Improving human cancer therapy through the evaluation of pet dogs. Nat Rev Cancer. (2020) 20:727–42. doi: 10.1038/s41568-020-0297-3, PMID: 32934365

[55] Tausch C Baege A Rageth C. Mapping lymph nodes in cancer management - role of (99m)Tc-tilmanocept injection. Onco Targets Ther. (2014) 7:1151–8. doi: 10.2147/OTT.S50394, PMID: 25028560 PMC4077853

[56] Surasi DS O'Malley J Bhambhvani P. 99mTc-Tilmanocept: a novel molecular agent for lymphatic mapping and sentinel lymph node localization. J Nucl Med Technol. (2015) 43:87–91. doi: 10.2967/jnmt.115.155960, PMID: 25956693

[57] Mwagiru D Shivashankar P Wong E Farlow D Cambden B Abdul-Razak M. Tilmanocept as a novel tracer for lymphatic mapping and sentinel lymph node biopsy in melanoma and oral cancer. ANZ J Surg. (2022) 92:2607–12. doi: 10.1111/ans.17868, PMID: 35848587 PMC9796895

[58] Azad AK Schlesinger LSM. Receptor (CD206)-mediated imaging in sentinel lymph node localization. *Clin Transl*. Imaging. (2015) 3:237–45. doi: 10.1007/s40336-015-0117-z

[59] Vera DR Wallace AM Hoh CK Mattrey RF. A synthetic macromolecule for sentinel node detection: 99mTc-DTPA-Mannosyl-dextran. J Nucl Med. (2001) 42:951–9. PMID: 11390562

[60] Reddy RA Moon AS Chow S Heilbroner L Howitt B Diver E. Technetium Tc 99m tilmanocept fails to detect sentinel lymph nodes in endometrial cancer. Gynecol Oncol Rep. (2022) 43:101054. doi: 10.1016/j.gore.2022.101054, PMID: 35958955 PMC9361318

[61] Hernot S van Manen L Debie P Mieog JSD Vahrmeijer AL. Latest developments in molecular tracers for fluorescence image-guided cancer surgery. Lancet Oncol. (2019) 20:e354–67. doi: 10.1016/S1470-2045(19)30317-1, PMID: 31267970

[62] Monteiro LN Rodrigues MA Gomes DA Salgado BS Cassali GD. Tumour-associated macrophages: relation with progression and invasiveness, and assessment of M1/M2 macrophages in canine mammary tumours. Vet J. (2018) 234:119–25. doi: 10.1016/j.tvjl.2018.02.016 29680383

[63] Belluco S Sammarco A Sapin P Lurier T Marchal T. FOXP3, CD208, and CD206 expression in canine cutaneous histiocytoma. Vet Pathol. (2020) 57:599–607. doi: 10.1177/0300985820941818, PMID: 32783525

[64] Stroup SP Kane CJ Farchshchi-Heydari S James CM Davis CH Wallace AM. Preoperative sentinel lymph node mapping of the prostate using PET/CT fusion imaging and Ga-68-labeled tilmanocept in an animal model. Clin Exp Metastasis. (2012) 29:673–80. doi: 10.1007/s10585-012-9498-9, PMID: 22714690

[65] Joshi BP Wang TD. Targeted optical imaging agents in Cancer: focus on clinical applications. Contrast Media Mol Imaging. (2018) 2018:2015237–19. doi: 10.1155/2018/2015237, PMID: 30224903 PMC6129851

[66] Boogerd LSF Hoogstins CES Gaarenstroom KN de Kroon CD Beltman JJ Bosse T. Folate receptor-α targeted near-infrared fluorescence imaging in high-risk endometrial cancer patients: a tissue microarray and clinical feasibility study. Oncotarget. (2017) 9:791–801. doi: 10.18632/oncotarget.23155 29416655 PMC5787511

[67] Randall LM Wenham RM Low PS Dowdy SC Tanyi JL. A phase II, multicenter, open-label trial of OTL38 injection for the intra-operative imaging of folate receptor-alpha positive ovarian cancer. Gynecol Oncol. (2019) 155:63–8. doi: 10.1016/j.ygyno.2019.07.010, PMID: 31362825

[68] Hoogstins CES Boogerd LSF Gaarenstroom KN de Kroon CD Beltman JJ Trimbos JBMZ. Feasibility of folate receptor-targeted intraoperative fluorescence imaging during staging procedures for early ovarian cancer. Eur J Gynaecol Oncol. (2019) 40:203–8. doi: 10.12892/ejgo4412.2019

[69] Kashiwagi S Choi HS. Ovarian cancer-targeted near-infrared fluorophores for fluorescence-guided surgery. Ann Transl Med. (2023) 11:274. doi: 10.21037/atm-22-6455, PMID: 37082670 PMC10113083

[70] Dindere ME Tanca A Rusu M Liehn EA Bucur O. Intraoperative tumor detection using Pafolacianine. Int J Mol Sci. (2022) 23:12842. doi: 10.3390/ijms232112842, PMID: 36361630 PMC9658182

[71] Dhawan D Ramos-Vara JA Naughton JF Cheng L Low PS Rothenbuhler R. Targeting folate receptors to treat invasive urinary bladder cancer. Cancer Res. (2013) 73:875–84. doi: 10.1158/0008-5472.CAN-12-2101 23204225

[72] Keating JJ Runge JJ Singhal S Nims S Venegas O Durham AC. Intraoperative near-infrared fluorescence imaging targeting folate receptors identifies lung cancer in a large-animal model. Cancer. (2017) 123:1051–60. doi: 10.1002/cncr.30419, PMID: 28263385 PMC5341137

[73] Gao RW Teraphongphom N de Boer E van den Berg NS Divi V Kaplan MJ. Safety of panitumumab-IRDye800CW and cetuximab-IRDye800CW for fluorescence-guided surgical navigation in head and neck cancers. Theranostics. (2018) 8:2488–95. doi: 10.7150/thno.24487, PMID: 29721094 PMC5928904

[74] Knapp DW. Urinary bladder cancer in dogs, a naturally occurring model for cancer biology and drug development. ILAR J. (2014) 55:100–18. doi: 10.1093/ilar/ilu018, PMID: 24936033

[75] Bergkvist GT Argyle DJ Pang LY Muirhead R Yool DA. Studies on the inhibition of feline EGFR in squamous cell carcinoma: enhancement of radiosensitivity and rescue of resistance to small molecule inhibitors. Cancer Biol Ther. (2011) 11:927–37. doi: 10.4161/cbt.11.11.15525, PMID: 21464610

[76] Pang LY Bergkvist GT Cervantes-Arias A Yool DA Muirhead R Argyle DJ. Identification of tumour initiating cells in feline head and neck squamous cell carcinoma and evidence for gefitinib induced epithelial to mesenchymal transition. Vet J. (2012) 193:46–52. doi: 10.1016/j.tvjl.2012.01.009, PMID: 22342216

[77] McCleese JK Bear MD Kulp SK Mazcko C Khanna C London CA. Met interacts with EGFR and Ron in canine osteosarcoma. Vet Comp Oncol. (2013) 11:124–39. doi: 10.1111/j.1476-5829.2011.00309.x, PMID: 22235915 PMC3969615

[78] Gama A Gärtner F Alves A Schmitt F. Immunohistochemical expression of epidermal growth factor receptor (EGFR) in canine mammary tissues. Res Vet Sci. (2009) 87:432–7. doi: 10.1016/j.rvsc.2009.04.016, PMID: 19464036

[79] Hassan BB Elshafae SM Supsavhad W Simmons JK Dirksen WP Sokkar SM. Feline mammary cancer. Vet Pathol. (2017) 54:32–43. doi: 10.1177/0300985816650243, PMID: 27281014 PMC7212821

[80] Selvarajah GT Verheije MH Kik M Slob A Rottier PJM Mol JA. Expression of epidermal growth factor receptor in canine osteosarcoma: association with clinicopathological parameters and prognosis. Vet J. (2012) 193:412–9. doi: 10.1016/j.tvjl.2012.02.009, PMID: 22436430

[81] Krishnan G van den Berg NS Nishio N Juniper G Pei J Zhou Q. Metastatic and sentinel lymph node mapping using intravenously delivered Panitumumab-IRDye800CW. Theranostics. (2021) 11:7188–98. doi: 10.7150/thno.55389, PMID: 34158844 PMC8210603

[82] Nishio N van den Berg NS van Keulen S Martin BA Fakurnejad S Teraphongphom N. Optical molecular imaging can differentiate metastatic from benign lymph nodes in head and neck cancer. Nat Commun. (2019) 10:5044. doi: 10.1038/s41467-019-13076-7, PMID: 31695030 PMC6834597

[83] Heath CH Deep NL Sweeny L Zinn KR Rosenthal EL. Use of panitumumab-IRDye800 to image microscopic head and neck cancer in an orthotopic surgical model. Ann Surg Oncol. (2012) 19:3879–87. doi: 10.1245/s10434-012-2435-y, PMID: 22669455 PMC3595117

[84] Zhang RR. Beyond the margins: real-time detection of cancer using targeted fluorophores. Nat Rev Clin Oncol. (2017) 14:347–64. doi: 10.1038/nrclinonc.2016.212, PMID: 28094261 PMC5683405

[85] Favril S. Clinical use of organic near-infrared fluorescent contrast agents in image-guided oncologic procedures and its potential in veterinary oncology. Vet Rec. (2018) 183:354–4. doi: 10.1136/vr.104851 29705740

[86] Chen F Madajewski B Ma K Karassawa ZD Stambuk H Turker MZ. Molecular phenotyping and image-guided surgical treatment of melanoma using spectrally distinct ultrasmall core-shell silica nanoparticles. Sci Adv. (2019) 5:eaax5208. doi: 10.1126/sciadv.aax5208, PMID: 31840066 PMC6892625

[87] Phillips E Penate-Medina O Zanzonico PB Carvajal RD Mohan P Ye Y. Clinical translation of an ultrasmall inorganic optical-PET imaging nanoparticle probe. Sci Transl Med. (2014) 6:260ra149. doi: 10.1126/scitranslmed.3009524, PMID: 25355699 PMC4426391

[88] Bradbury MS Phillips E Montero PH Cheal SM Stambuk H Durack JC. Clinically-translated silica nanoparticles as dual-modality cancer-targeted probes for image-guided surgery and interventions. Integr Biol Quant Biosci Nano Macro. (2013) 5:74–86. doi: 10.1039/c2ib20174g, PMID: 23138852 PMC4428677

[89] Aron A Zavaleta C. Current and developing lymphatic imaging approaches for elucidation of functional mechanisms and disease progression. Mol Imaging Biol. (2023) 26:1–16. doi: 10.1007/s11307-023-01827-4, PMID: 37195396 PMC10827820

[90] Zanoni DK Stambuk HE Madajewski B. Use of ultrasmall core-shell fluorescent silica nanoparticles for image-guided sentinel lymph node biopsy in head and neck melanoma: a nonrandomized clinical trial. JAMA Netw Open. (2021) 4:211936. doi: 10.1001/jamanetworkopen.2021.1936, PMID: 33734415 PMC7974643

[91] Favril S Brioschi C Vanderperren K Abma E Stock E Devriendt N. Preliminary safety and imaging efficacy of the near-infrared fluorescent contrast agent DA364 during fluorescence-guided surgery in dogs with spontaneous superficial tumors. Oncotarget. (2020) 11:2310–26. doi: 10.18632/oncotarget.27633, PMID: 32595830 PMC7299531

[92] Wenk CH Ponce F Guillermet S Tenaud C Boturyn D Dumy P. Near-infrared optical guided surgery of highly infiltrative fibrosarcomas in cats using an anti-αvß3 integrin molecular probe. Cancer Lett. (2013) 334:188–95. doi: 10.1016/j.canlet.2012.10.041, PMID: 23200675

[93] Ludwig BS Kessler H Kossatz S Reuning U. RGD-binding Integrins revisited: how recently discovered functions and novel synthetic ligands (re-)shape an ever-evolving field. Cancers. (2021) 13:1711. doi: 10.3390/cancers13071711, PMID: 33916607 PMC8038522

[94] Singh B Rawlings N Kaur A. Expression of integrin alphavbeta3 in pig, dog and cattle. Histol Histopathol. (2001) 16:1037–46. doi: 10.14670/HH-16.1037, PMID: 11642723

[95] Rawlings NG Simko E Bebchuk T Caldwell SJ Singh B. Localization of integrin alpha(v)beta3 and vascular endothelial growth factor receptor-2 (KDR/Flk-1) in cutaneous and oral melanomas of dog. Histol Histopathol. (2003) 18:819–26. doi: 10.14670/HH-18.819, PMID: 12792894

[96] Banerji S Ni J Wang SX Clasper S Su J Tammi R. LYVE-1, a new homologue of the CD44 glycoprotein, is a lymph-specific receptor for hyaluronan. J Cell Biol. (1999) 144:789–801. doi: 10.1083/jcb.144.4.789, PMID: 10037799 PMC2132933

[97] Breiteneder-Geleff S Soleiman A Horvat R Amann G Kowalski H Kerjaschki D. Podoplanin--a specific marker for lymphatic endothelium expressed in angiosarcoma. Verh Dtsch Ges Pathol. (1999) 83:270–5. PMID: 10714221

[98] Licha K Debus N Emig-Vollmer S Hofmann B Hasbach M Stibenz D. Optical molecular imaging of lymph nodes using a targeted vascular contrast agent. J Biomed Opt. (2005) 10:041205. doi: 10.1117/1.2007967 16178629

[99] Laakkonen P Porkka K Hoffman JA Ruoslahti E. A tumor-homing peptide with a targeting specificity related to lymphatic vessels. Nat Med. (2002) 8:751–5. doi: 10.1038/nm720, PMID: 12053175

[100] Yang X Wang Z Zhang F Zhu G Song J Teng GJ. Mapping sentinel lymph node metastasis by dual-probe optical imaging. Theranostics. (2017) 7:153–63. doi: 10.7150/thno.17085, PMID: 28042324 PMC5196893

